# Advances in Osteosarcoma

**DOI:** 10.1007/s11914-023-00803-9

**Published:** 2023-06-17

**Authors:** Isidora Panez-Toro, Javier Muñoz-García, Jorge W. Vargas-Franco, Axelle Renodon-Cornière, Marie-Françoise Heymann, Frédéric Lézot, Dominique Heymann

**Affiliations:** 1 Nantes Université, CNRS, UMR6286, US2B, Biological Sciences and Biotechnologies unit, 44322 Nantes, France; 2 Institut de Cancérologie de l’Ouest, Tumor Heterogeneity and Precision Medicine Laboratory, 44805 Saint-Herblain, France; 3 University of Antioquia, Department of Basic Studies, Faculty of Odontology, Medellin, Colombia; 4 Sorbonne Université, INSERM UMR933, Hôpital Trousseau (AP-HP), 75012 Paris, France; 5 University of Sheffield, Medical School, Department of Oncology and Metabolism, S10 2RX, Sheffield, UK

**Keywords:** Osteosarcoma, Immunotherapy, Tumor targeted therapy, Immune checkpoint inhibitor, Tumor microenvironment, Personalized medicine

## Abstract

**Purpose of Review:**

This article gives a brief overview of the most recent developments in osteosarcoma treatment, including targeting of signaling pathways, immune checkpoint inhibitors, drug delivery strategies as single or combined approaches, and the identification of new therapeutic targets to face this highly heterogeneous disease.

**Recent Findings:**

Osteosarcoma is one of the most common primary malignant bone tumors in children and young adults, with a high risk of bone and lung metastases and a 5-year survival rate around 70% in the absence of metastases and 30% if metastases are detected at the time of diagnosis. Despite the novel advances in neoadjuvant chemotherapy, the effective treatment for osteosarcoma has not improved in the last 4 decades. The emergence of immunotherapy has transformed the paradigm of treatment, focusing therapeutic strategies on the potential of immune checkpoint inhibitors. However, the most recent clinical trials show a slight improvement over the conventional polychemotherapy scheme.

**Summary:**

The tumor microenvironment plays a crucial role in the pathogenesis of osteosarcoma by controlling the tumor growth, the metastatic process and the drug resistance and paved the way of new therapeutic options that must be validated by accurate pre-clinical studies and clinical trials.

## Introduction

Benign and malignant primary bone tumors are classified based on the tissue type from which the tumor started, such as bone-forming, cartilage-forming, connective, vascular, and idiopathic tumors. Malignant primary bone tumors, also called bone sarcomas, are a group of rare malignancies that originates in the skeletal system, with a prevalence for the long bones but not exclusively. Bone sarcomas are highly aggressive, and patients suffer from pain, swelling and fractures [1, 2]. These tumors are difficult to treat, are characterized by many histological variations, and present many clinical difficulties when identifying resolutive therapies [1]. The three main entities of bone sarcomas are osteosarcomas (OS), Ewing sarcomas, and chondrosarcomas [1, 3].

OS are the most prevalent malignant bone tumors in adolescents and young adults [4–9]. OS are mainly located in the metaphysis of long bones, especially the distal femur, proximal tibia, and humerus [4, 7, 9]. They derive from primitive mesenchymal cells and are characterized by immature osteoid extracellular matrix formation related to bone resorption mediated by activated osteoclasts [4, 6–10]. The biological origin of OS remains unclear, although multiple factors may be responsible for the disease, including genetic mutations (e.g., *Rb*, *p53*) and an immunosuppressive microenvironment which fuels tumor development [10–12]. OS development may also be related to the “seed and soil” theory initially proposed by Sir Steven Paget at the end of the nineteenth century. OS cells grow in a permissive local microenvironment which modulates their behavior and facilitates all steps in tumor development (e.g., proliferation/quiescence, invasion/migration, drug resistance) [4, 13–15] and contributes to their intrinsic heterogeneity [16, 17••]. The lung parenchyma is the most common metastatic site in OS, and metastatic foci are frequently associated with a poor clinical outcome [7, 9, 18].

The conventional treatment for OS is based on a sequential approach that combines chemotherapy and surgery [4]. Due to the particular radioresistance of OS, radiotherapy is only proposed for tumors in high-risk locations, or to reduce the risk of recurrence after surgery [7–9, 18–20]. Despite the increase in clinical trials in the last 4 decades, cure rates for OS have not improved. Non-specific targeting therapies thus show poor therapeutic effects with side effects at high doses [20].

This review aims to summarize and discuss the main recent advances in OS therapeutic approaches.

## Signaling Pathways

### RANK Pathway

The Receptor Activator of Nuclear Factor κB (RANK)/RANK Ligand (RANKL)/Osteoprotegerin (OPG) axis is the main molecular triad that drives osteoclastogenesis and bone resorption [21]. RANKL induces osteoclast precursor fusion and differentiation through its binding to RANK, and OPG is a decoy receptor blocking the interaction between RANK and RANKL. Osteoclasts and OS cells cooperate through reciprocal molecular regulation that contributes to the pathogenesis of OS and associated bone resorption [22]. However, the role of osteoclasts in the biology of OS remains controversial. At the initiation stage of the disease, osteoclast differentiation and activities stimulated by OS cells fuel OS cell proliferation though the release of degradation products of the extracellular matrix. On the contrary, these multinucleated cells may play opposing functions with beneficial activities when the tumor is installed and the metastatic process is initiated [23, 24]. In addition, OS cells express RANK and may be a potential target for anti-RANKL therapies, even the RANK expression by OS cells remains controversial [25–28]. Pre-clinical observations strengthen the potential therapeutic interest of RANKL blockade in OS by using various approaches (anti-RANKL antibodies, siRNAs, OPG) [29–32]. A clinical trial (ClinicalTrials.gov Identifier: NCT02470091) is currently ongoing to evaluate the therapeutic benefit of denosumab in OS. The estimated completion date is September 2023.

**Fig. 1:**
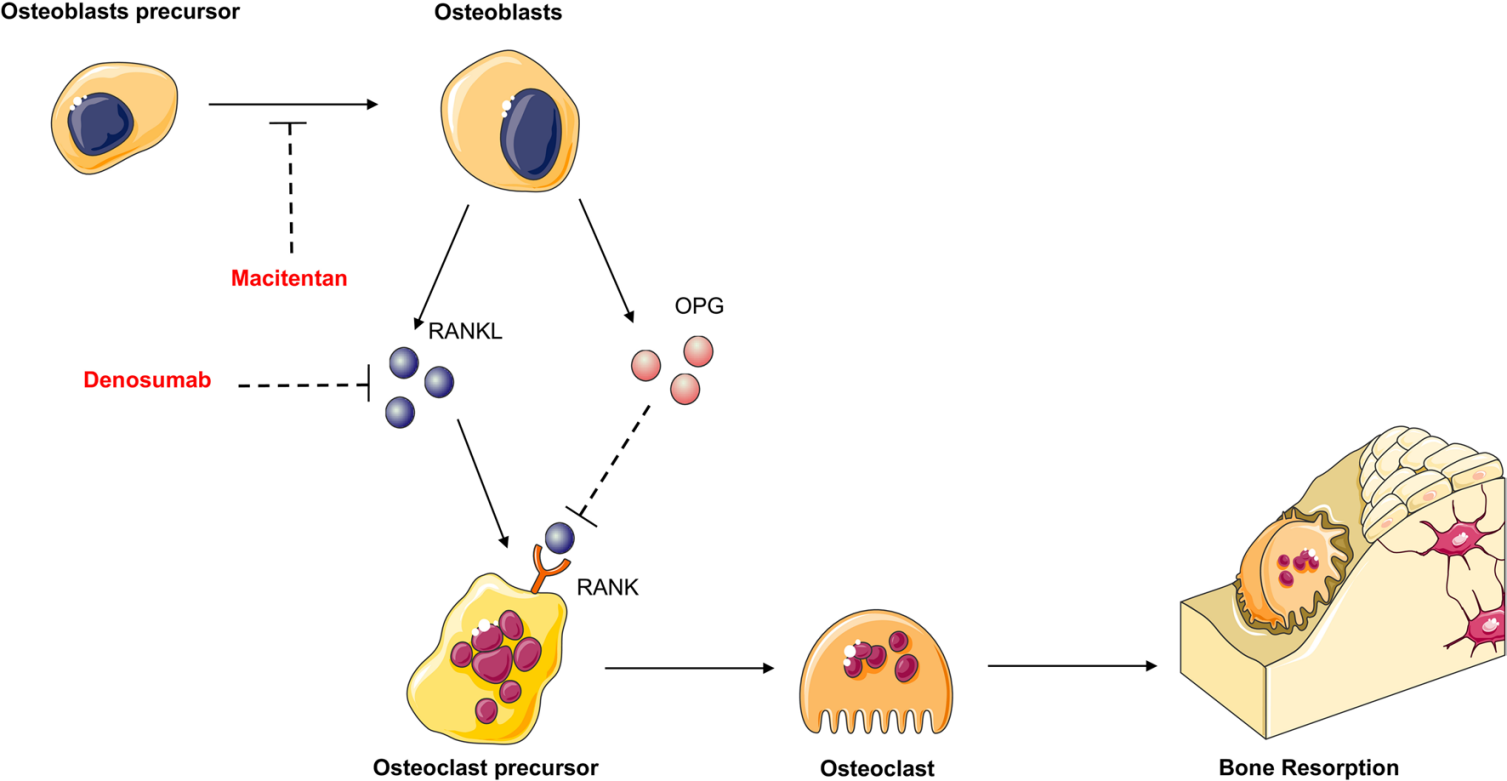
New therapeutic approaches based on RANK/RANKL/OPG signaling. The RANKL/RANK/OPG pathway controls osteoclastogenesis and orchestrates bone remodeling. RANKL expressed at the surface of osteoblasts and in a soluble form binds to RANK expressed by osteoclast precursor membranes leading to osteoclast differentiation and bone resorption. OPG acts as a decoy receptor in this system and interrupts RANK-RANKL binding and signaling, and consequently inhibits bone resorption. Combined anti-RANKL therapy with macitentan, an inhibitor of both ETA-B endothelin receptors, was associated with a decrease in lung metastases, as well as a bone protective reaction in a preclinical OS murine model

Anti-RANKL therapies combined with other approaches have recently been assessed. The combination of anti-RANK therapy with conventional chemotherapeutic agents does not appear recommended based on in vitro investigations showing reduced cytotoxic activity of doxorubicin in the presence of denosumab [33]. The endothelin pathway (the ligands ET1-3 and their two receptors ETA-B) is associated with bone formation by targeting osteoblast differentiation and function [34]. Macitentan is an inhibitor of both ETA-B endothelin receptors [35]. Despite the absence of a direct effect on tumor growth, the combination of macitentan with RANKL blockade resulted in a decrease in lung metastases as well as a bone protective action in a preclinical OS murine model (Fig. 1) [35].

### Wnt Signaling

**Fig. 2:**
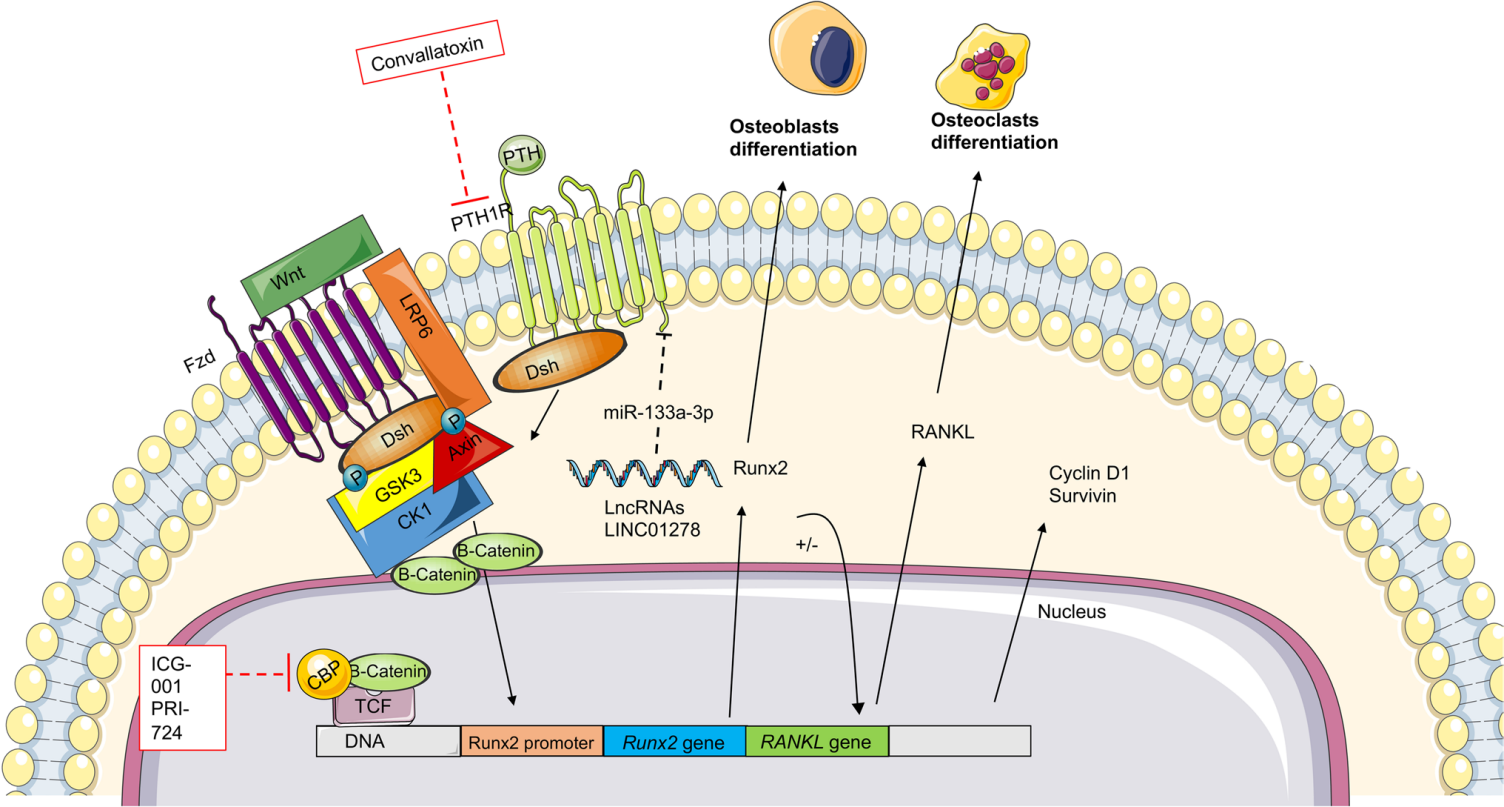
Targeting of Wnt signaling in OS. OS is characterized by heterogeneity in the expression of Wnt ligands and receptors. Due to their over-activation in OS, the transcriptional targets of Wnt/β-catenin are associated with pathways responsible for OS progression. Among these pathways modulated by Wnt/β-catenin, cyclin D1 and survivin are involved in cell proliferation, runx2 in osteogenic differentiation, and RANKL in osteoclast activation. The ICG-001 derivative PRI-724 inhibits cell proliferation due to the blockade of the CREB binding protein (CBP)/β-catenin complex formation in vitro. The parathyroid hormone (PTH) and its parathyroid hormone receptor 1 (PTHR1) activate the Wnt/β-catenin pathway in OS cells. Long non-coding RNA LINC01278 expressed in the cytoplasm of OS cells blocks miR-133a-3p, a tumor inhibitor molecule of PTHR1, promoting the upregulation and release of PTHR1, and may serve as an oncogene in OS development. Convallatoxin downregulates the expression of PTHR1

The Wnt signaling pathway is an evolutionarily conserved pathway responsible for cell fate determination, stem cell replication, survival, differentiation, calcium homeostasis, cell polarity, and osteogenic differentiation [36]. This signaling pathway is mediated by Wnt ligands, membrane receptors, and co-receptors such as phosphoprotein dishevelled (Dsh) and low-density lipoprotein receptor-related protein (LRP) [37]. Due to their overactivation in OS, the most studied ligand is the Wnt/β-catenin pathway [36, 38]. Even the expression level of the Wnt ligands and receptors exhibited high heterogeneity in OS, and the transcriptional targets of Wnt/β-catenin are associated with pathways responsible for OS progression, including genes involved in cell proliferation, such as *MYC* and *cyclin D1*, *runx2* in osteogenic differentiation and *RANKL* in osteoclastogenesis and osteoclast activation [37]. Wnt signaling induces very complex effects with dual activities in OS. The ICG-001 derivative PRI-724, an inhibitor of the CREB binding protein (CBP)/β-catenin complex formation that downregulates Wnt/β-catenin-mediated transcription, showed an ability to slow down the migration of human 143B OS cells, as well as to decrease human SJSA-1 OS cell proliferation and invasion in vitro. This effect was due to the reduction in cyclin D1 and survivin protein levels, respectively, regulating cell proliferation and cell division. In addition, PRI-724 inhibited the clonogenic ability of 143B and SJSA-1 cells (Fig. 2) [36].

On the contrary, deleterious activities were assigned to Wnt/β-catenin signaling. More specifically, its overexpression may contribute to OS development and the renewal of cancer stem cells [19, 36]. In three human OS cell lines, KHOS, MG63, and 143B, ICG-001 inhibits OS in vitro cell proliferation but increases their migration. Moreover, ICG-001 also increased the metastatic dissemination to the lungs in a preclinical mouse model [38]. Although a clinical phase I/IIa study concluded that PRI-724 is tolerated by patients with liver fibrosis (ClinicalTrials.gov Identifier: NCT03620474), its efficacy in OS has not been studied yet [39].

The parathyroid hormone and its parathyroid hormone receptor 1 (PTHR1) have been associated with the malignant progression of OS by activating the Wnt/β-catenin pathway [40, 41]. Long non-coding RNA can act as a regulator of tumor-suppressive or oncogenic genes by mediating the proliferation, apoptosis, migration, and metastasis of tumor cells [42]. In OS, long non-coding RNAs (lncRNAs) function as endogenous sponges for miRNAs. LINC01278, an lncRNA, was expressed in the cytoplasm of OS cells which acted as a sponge for miR-133a-3p, a tumor inhibitor molecule, and favored OS progression [43]. LINC01278, another lncRNA that acts as a sponge for miR-133a-3p, promoted the release and upregulation of PTHR1 and has been proposed as serving as an oncogene in OS development (Fig. 2) [43].

Cardiac glycoside has been reported as a potent anti-tumor molecule as it selectively inhibits tumor cell proliferation [41]. Convallatoxin is a natural cardiac glycoside found in *Convallaria majalis*, a plant known as Lily of the valley [44]. It has been reported that convallatoxin promotes apoptosis and inhibits proliferation and angiogenesis in colorectal cancer cells [45]. In OS, convallatoxin was capable of inhibiting MG63 and U2OS OS cell proliferation, migration, and invasion by reducing expressions of MMP2 and MMP9 gelatinases, which are involved in the degradation and remodeling of the extracellular matrix [41]. In addition, convallatoxin increases the protein expression of collagen 1, osteopontin, osteocalcin, and Runx2, and decreases RANKL protein expressions, which promote osteogenic differentiation of OS cells. Interestingly, convallatoxin inhibits OS cell proliferation, migration, and invasion by suppressing PTHR1 expression and Wnt/β-catenin pathways dose-dependently (Fig. 2) [41]. Despite its dual effects, the Wnt/β-catenin pathway might be a promising treatment for OS.

### Ferroptosis and Cell Death Pathway

Ferroptosis is a programmed cell death pathway associated with iron overload, leading to reactive oxygen species (ROS) accumulation, disruption of redox homeostasis and lipid peroxidation reactions that promote cell death [46]. To date, several ferroptosis pathways have been described, such as regulatory axis GSH-GPX4, iron metabolism pathway, lipid peroxidation pathway, dihydroorotate dehydrogenase (DHODH), voltage-dependent anion channels, and the GTP cyclohydrolase 1 (GCH1)-tetrahydrobiopterin (BH4) pathway [46]. Focusing on the GSH-GPX4 pathway, Xiang et al*.* have identified glutathione peroxidase 4 (GPX4) as a potential inhibitor of the cytotoxic effects of ferroptosis inducers through a synergistic effect with glutathione (GSH). Moreover, knockdown experiments of both GPX4 and its inhibitor RSL3 may induce ferroptosis [46]. In this context, insufficient levels of GSH would reduce GXP4 efficacy, leading to the accumulation of lipid peroxides which also cause ferroptosis. Interestingly, activation of the p53 gene could exert its tumor suppressor effect by downregulating the expression of SLC7A11. SLC7A11 is a cystine/glutamate antiporter solute carrier family 7 member 11 that promotes cystine uptake and glutathione biosynthesis. SLC7A11 regulates ferroptosis by controlling the synthesis of GSH, affecting the GSH-GXP4 axis [46, 47]. Using glutaminase inhibitors on tumor cells with high expression of SLC7A11 increases cell mortality in an independent GSH-GPX4 pathway [48]. SLC7A11 made tumor cells highly dependent on glucose and glutamine [47]. The nutrient dependence caused by SLC7A11 enhances ferroptosis in tumor cells, and this pathway may solve chemoresistance in OS.

**Fig. 3:**
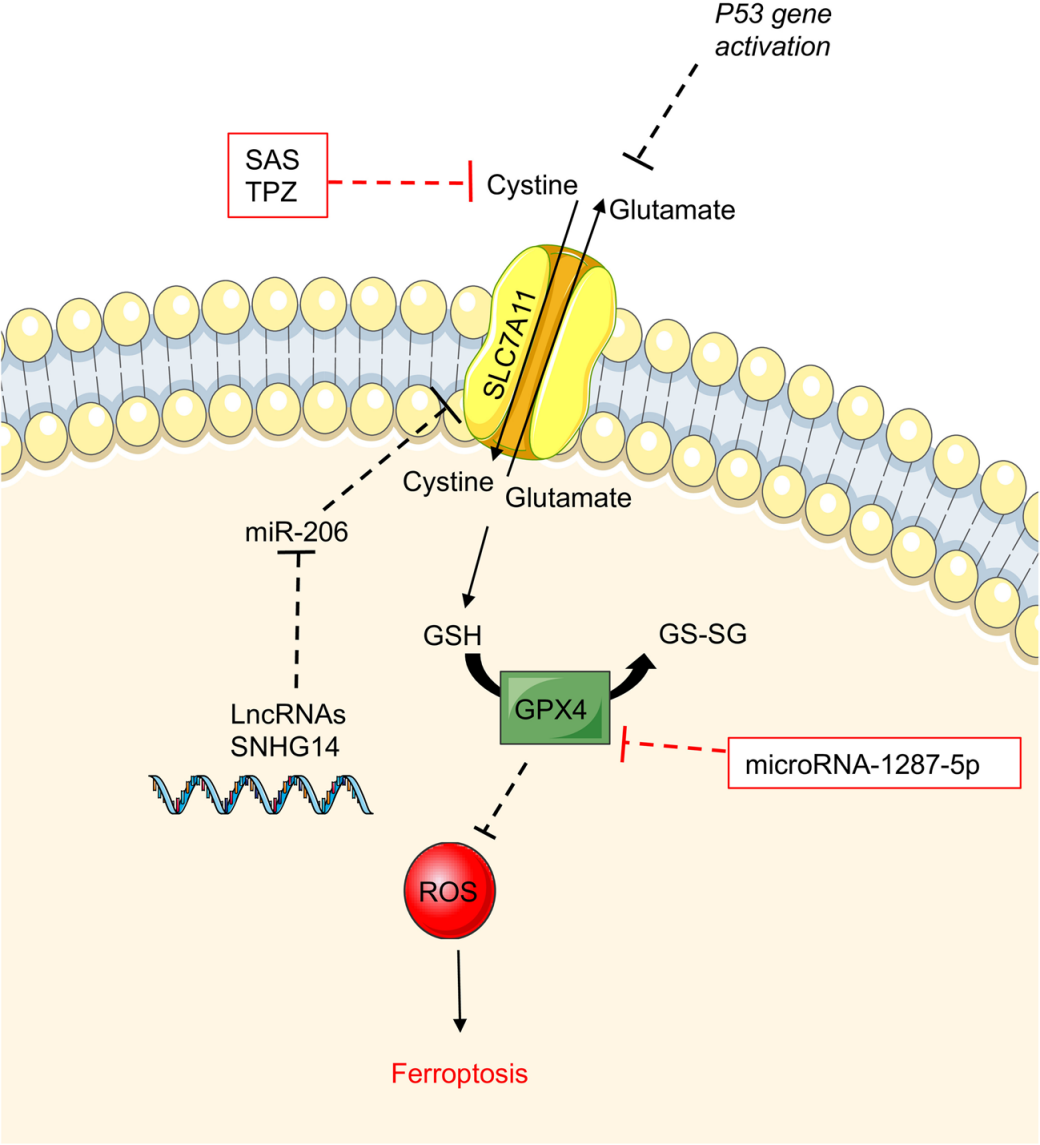
Ferroptosis in OS cells. Ferroptosis is a programmed cell death pathway related to reactive oxygen species (ROS) accumulation. SLC7A11 is a cystine/glutamate antiporter that regulates ferroptosis by controlling the synthesis of GSH, affecting the GSH-GXP4 axis. In OS cells, activation of the p53 gene downregulates the expression of SLC7A11 leading to a tumor suppressor effect. The lncRNA SNHG14 is upregulated in OS cells, downregulates expression of miR-206, affecting the ferroptosis inhibitor SLC7A11, preventing ferroptosis. Sulfasalazine (SAS) and tirapazamine (TPZ) are inhibitors of the cystine/glutamate system affecting GPx4 activity and the consequent accumulation of ROS. microRNA-1287-5p inhibits the inhibition of GPX4, enhancing OS cell death via ferroptosis

Recent new regulation pathways have been described. The lncRNA SNHG14 is upregulated in OS cell lines contributing to treatment resistance by suppressing ferroptosis. The resistance disappeared using ferrostatin-1, a ferroptosis inhibitor. The lncRNA SNHG14 downregulated expression of miR-206, affecting the ferroptosis inhibitor SLC7A11 and preventing cells from undergoing ferroptosis (Fig. 3) [49].

Sulfasalazine (SAS) is an FDA-approved drug for rheumatoid arthritis and inflammatory bowel disease. It is an inhibitor of the cystine/glutamate system affecting GPx4 activity and induces ferroptosis in OS cells. The combination of SAS with iron resulted in a more effective treatment (Fig. 3) [50]. Tirapazamine (TPZ) is an anticancer drug targeting hypoxic tumor cells by increasing intracellular ROS and may inhibit the proliferation and migration of OS cells. Like SAS, inhibiting SLC7A11 induces ferroptosis [51]. In addition, it has been demonstrated that microRNA-1287-5p inhibits OS cells by ferroptosis via inhibition of GPX4 (Fig. 3) [52]. Overall, targeting ferroptosis may provide a new therapeutic option in OS [53].

## Immune Checkpoint Inhibitors and Immunotherapy in OS

The efficiency of a cancer treatment remains a challenging issue due to potential drug toxicity and the chemoresistance properties of tumor cells [54]. Tumor cells are capable of stimulating the tumor microenvironment (TME) to suppress the antitumor immune system, leading to tumor drug resistance [54]. The development of personalized precision medicine in recent decades, including immunotherapies, has shown tremendous benefits in treating malignant tumors [54, 55]. As illustrated by the successful treatment of metastatic melanoma with ipilimumab, several immune checkpoint inhibitors have been approved by the FDA [55, 56]. The main list of immune checkpoint inhibitors includes programmed death-1 (PD-1), death-ligand 1 (PD-L1), and cytotoxic T lymphocyte-associated protein 4 (CTLA4) [54] and they are all therapeutic options in OS**.**

### PD-1

**Fig. 4:**
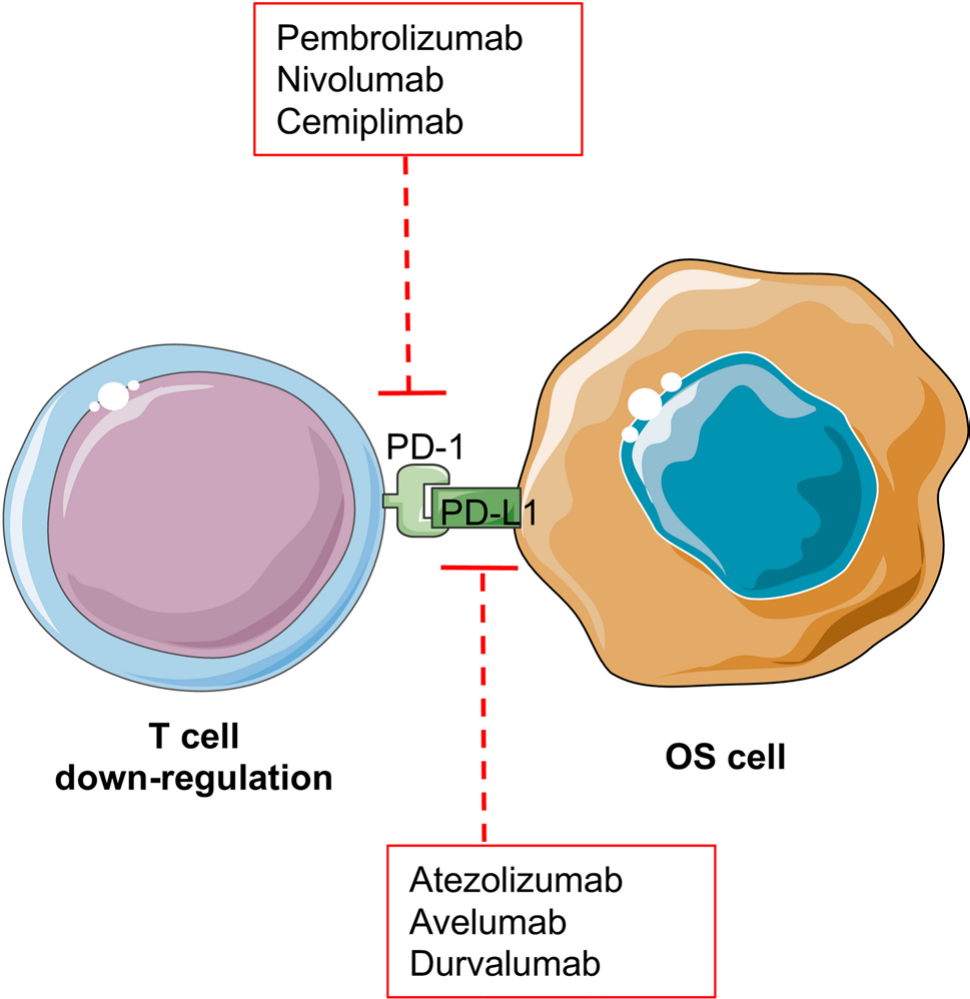
PD-1 in OS cells. PD-1 is a transmembrane protein that acts as an immune checkpoint receptor that is expressed in CD4^+^ and CD8^+^ T cells. The interaction with their ligand PD-L1 downregulates T cells leading to an adaptive immune tolerance. PD-1 inhibitors such as pembrolizumab, nivolumab, and cemiplimab, and PD-L1 inhibitors such as atezolizumab, avelumab, and durvalumab are currently approved by the FDA

PD-1, a transmembrane protein, acts as an immune checkpoint receptor that is expressed at the surface of CD4^+^ and CD8^+^ T cells, dendritic cells, and macrophages. This immune checkpoint interacts with their ligands PD-L1 (B7-H1, CD274) and PD-L2 (B7-DC, CD273), which suppress T cell functions by inducing T cell exhaustion and downregulation, leading to adaptive immune tolerance (Fig. 4) [55, 57, 58].

The high PD-1 activity in the tissue microenvironment can suppress tumor immunosurveillance, and their expression level correlates with poor patient prognosis [57–59]. Monoclonal antibodies PD-1/PD-L1 were then developed to waive immunosuppressive signaling and local immunotolerance. PD-1 inhibitors such as pembrolizumab, nivolumab, and cemiplimab, and PD-L1 inhibitors such as atezolizumab, avelumab, and durvalumab, are currently approved by the FDA and EMEA (Fig. 4) [55, 57, 58]. Several PD-1 inhibitors decrease tumor lymphocyte infiltration as well as T regs and upregulate the cytolytic activity of CD8^+^ T cells [60]. Moreover, targeting PD-1 might induce tumor regression in pulmonary metastases in an OS mouse model by increasing the macrophage polarization of M1 versus M2 [61].

In a phase I/II clinical trial with young patients with relapsed solid tumors (ADVL1412, ClinicalTrials.gov Identifier: NCT02304458), nivolumab did not show any activity for the single agent in pediatric solid tumors. Also, their data demonstrate low PD-L1 expression levels and a decrease in infiltrating T cells [62]. Interestingly, pediatric patients with high mutation tumor burden showed a significant blockade of PD-1, highlighting the value of PD-1 inhibitors in improving the T cell response [62]. Pembrolizumab was tested in a multicenter phase II clinical trial to explore its safety and efficacy in patients with bone and soft-tissue sarcoma (SARC028, ClinicalTrials.gov Identifier: NCT02301039) [63]. This study demonstrated the limited effect of pembrolizumab with only one objective response out of 22 patients, which may be explained by the highly variable expression of PD-L1 in OS [63]. Recently, a phase II clinical trial based on pembrolizumab monotherapy in advanced OS did not show clinically relevant anti-tumor activity at 18 weeks of treatment. Of the 12 patients from the study, PD-L1 expression was positive in 1 patient, in whom a mixed response and tumor-intrinsic signaling through TGF-β and Wnt signaling activity were reduced. These findings may suggest that the lack of response to pembrolizumab in the other 11 OS patients might be due to the increase in TGF-β and Wnt signaling associated with an immunosuppressive tumor microenvironment with low T cell infiltration [64]. The limited therapeutic efficacy of pembrolizumab was confirmed [65] and studies combining it with conventional chemotherapy, which appears well tolerated (ClinicalTrials.gov Identifier: NCT02888665) [66], are warranted.

Better stratification of OS patients in anti-PD1 therapies is mandatory and there is a need to explore combination strategies based on molecular profiles associated with their response. Several biomarkers have been proposed to identify the predictability of response to PD-1 antagonists, including (i) > 1% of PD-L1^+^ tumor cells determined by immunohistochemistry, and (ii) microsatellite instability examination for the tumor cells deficient in mismatched DNA repair systems [58]. The first human, open-label, I/II phase study of cetrelimab (JNJ-63723283) in patients with advanced or refractory solid tumors showed a safety profile consistent with other anti-PD-1 antibodies reported in the literature. However, the prevalence of infusion-related reactions was higher compared to pembrolizumab and nivolumab reports even if the sample size was small [58] (ClinicalTrials.gov Identifier: NCT02908906). This study suggests standardization of microsatellite instability testing due to the high variability in patients as a means of improving reliability [58]. Furthermore, a recent study identified the tumor DNA methylation profiles as a predictive marker of the therapeutic response to anti-PD-1 immune checkpoint inhibitor monotherapy and may help with future patient stratification [67].

### CTLA-4

**Fig. 5:**
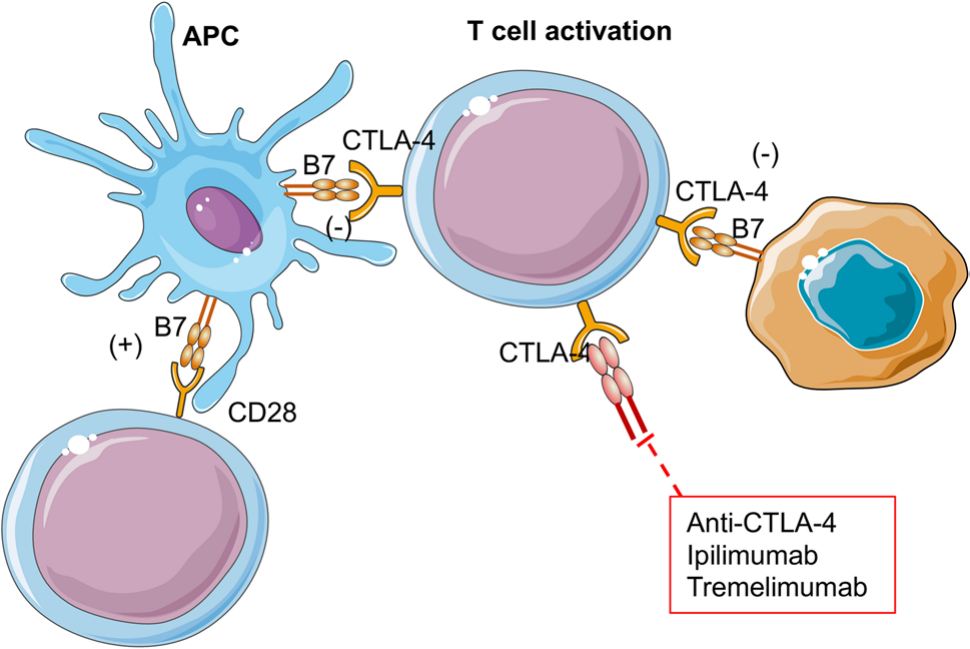
CTLA-4 in OS cells. The cytotoxic T cell lymphocyte antigen 4 (CTLA-4) is expressed on regulatory T cells and is a co-inhibitory receptor for CD28 for its B7 ligands during T cell activation. CTLA-4 is expressed in OS cells leading to immune system evasion. Ipilimumab, anti-CTLA-4 antibody, and tremelimumab (Anti-CTLA-4) were tested in clinical trials in combination with anti-PD-L1/PD-1 antibodies in sarcoma subtypes

The cytotoxic T cell lymphocyte antigen 4 (CTLA-4) is expressed on regulatory T cells. CTLA-4 is homologous to T cell co-stimulatory protein CD28 and is also a co-inhibitory receptor for T cell activation that competes with CD28 for its B7 ligands. During T cell activation, CD28 receptors on T cells bind to B7 ligands, such as CD80 and CD86, expressed in antigen-presenting cells (APC) and play a role as second activation signals for T cells [55, 57]. When CTLA-4 binds to B7 ligands, a co-inhibitory signaling pathway indirectly deprives T cells of activation. The lack of T cell activation leads tumor cells to evade the immune system [55]. CTLA-4 is expressed in OS cells, improving the capacity for immune system evasion (Fig. 5) [55].

Ipilimumab was assessed in a phase I clinical trial, conducted to determine the pharmacokinetic parameters, safety, and toxicity profiles in monotherapy in pediatric patients with solid tumors including OS [68]. Interestingly, even if no objective tumor regression was observed, patients who showed a break in immune tolerance identified by immune-related toxicities had increased overall survival.

Anti-CTL-4 therapy was combined with the anti-PD-L1 approach. Evaluation of the anti-tumor efficacy of combined nivolumab (anti-PD-L1) and ipilimumab (anti-CTLA-4) therapies in a metastatic OS patient resulted in complete stabilization of life-threatening retrocardiac lesions and progression of bone wounds and pleural metastases [69]. Two clinical trials are currently in progress in OS patients (ClinicalTrials.gov Identifiers: NCT02304458 and NCT02500797).

A single-center phase II trial (ClinicalTrials.gov Identifier: NCT02815995) based on combined treatment associating durvalumab (Anti-PD-1) and tremelimumab (Anti-CTLA-4) therapies in 56 patients with advanced or metastatic bone and soft-tissue sarcomas showed 49% progression-free survival at 12 weeks (Fig. 5). However, the combination appeared more active in specific soft-tissue sarcoma histological subtypes. Interestingly, biopsy with immune profiling of patients with alveolar soft-part sarcoma did not show a significant increase in tumor-associated immune cells, but rather a significant increase in cytotoxic CD3^+^CD8^+^ T cells and antigen-experienced CD3^+^CD8^+^PD-1^+^) T cells compared to the baseline. This observation suggests that individualized early assessment of each patient's expression for tumor-associated immune cells could be a more predictive tool than baseline biopsies [70]. Interestingly, some non-responders with an increase in the density of cytotoxic T-cells also had an increase in regulatory T cells [70]. This observation strengthens the role of the associated microenvironment that regulates and makes possible tumor growth and immune evasion. These results highlight the need for the research community to better understand the tumor environment in a spatial and temporal manner for each patient.

### TIM-3

**Fig. 6:**
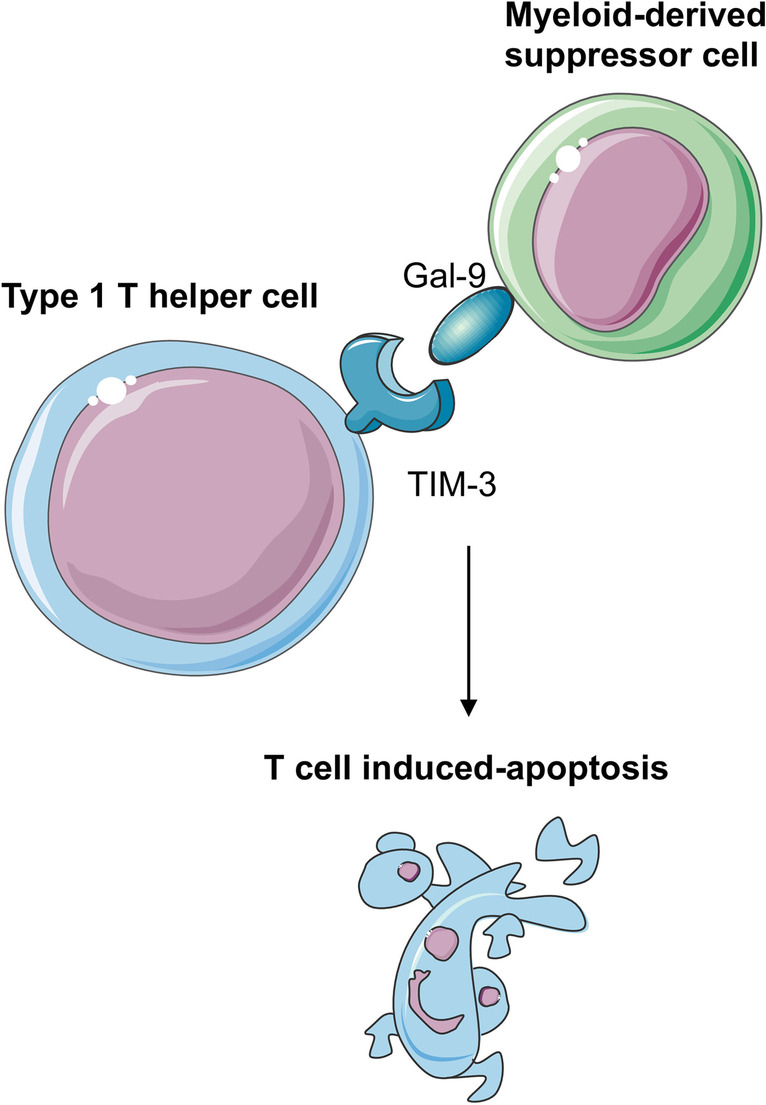
TIM-3 in OS tumors. The receptor T cell immunoglobulin and mucin-containing protein-3 (TIM-3) expressed on type 1 T helpers binds to their ligand galactin-9 (Gal-9), inducing Th1 apoptosis and tolerance of T cells in the OS tumor microenvironment

The receptor T cell immunoglobulin and mucin-containing protein-3 (TIM-3) is expressed on type 1 T helper (Th1), Th17, monocytes and macrophages [55, 57, 71]. When TIM-3 binds to their ligand galactin-9 (Gal-9), it acts as a downregulator of anti-tumor immunity by inducing Th1 apoptosis and tolerance of T cells (Fig. 6) [55, 71].

TIM-3 and Gal-9 are expressed in OS tumors, and their signaling pathway promotes apoptosis of CD4^+^ and CD8^+^ T cells in the tumor microenvironment [55]. Thus, expression of inhibitory checkpoints in the tumor microenvironment of OS pulmonary metastases, such as TIM-3, Lag-3, and IDO1, are associated with immunosuppression [72].

Immune checkpoint inhibitors benefit several malignant entities because of their high levels of immune infiltration in the tumor microenvironment. However, OS is described as a “cold tumor” with regard to poor local immune activation and cancer cell recognition, even in the presence of detectable immune cells in the tumor microenvironment [14, 73]. This immune tolerant environment may be explained by adaptive immune resistance mechanisms (higher expressions of PD-L1, CTLA-4, and IDO1) and the presence of myeloid-derived suppressor cells that inhibit T cell activation and enhance the immune evasion of OS [74•]. Overall, using immune checkpoint inhibitors (monotherapy or dual therapy) has limited clinical value. However, it is crucial to clarify the mechanisms of action associated with this immune response desert to reverse local immunity in OS.

## Role of the Tumor Microenvironment in OS Drug Resistance

Advanced OS has highly heterogeneous histological patterns and is associated with an immunosuppressive microenvironment, which comprises cancer-associated fibroblasts, tumor-associated macrophages, vascular and perivascular cells, mesenchymal stem cells, dendritic cells, neutrophils, and CD4^+^ and CD8^+^ tumor-infiltrating lymphocytes, FoxP3^+^ T regs, B lymphocytes, and NK cells [12, 17••, 75–77]. More recently, tumor-specific intracellular bacteria have been shown to enrich the tumor microenvironment of osteosarcoma [78••, 79]. The pulmonary metastatic microenvironment is characterized by a higher infiltration of T cells and relatively lower myeloid cells than primary bone tumors and higher expressions of inhibitory checkpoints, such as TIM-3, Lag-3, and IDO1 [72]. In addition, a specific extracellular matrix with collagens, laminins, cell receptors and integrins, matrix metalloproteinases, and heparinase plays a major role in OS development by controlling cell behavior. For instance, collagen produced by cancer-associated fibroblasts has a protective tumor effect by trapping T cells and downregulating the anti-tumor activity of tumor-infiltrating T cells [55]. Overall, these components in the tumor microenvironment play roles in OS development, invasion, metastases, and resistance.

Recent publications have underlined the contribution of mesenchymal stem cells in the proliferation and migration of OS cells and in the mechanisms of drug resistance [76, 80, 81]. OS cells coordinate the functional education of mesenchymal stem cells by secreting TGF-β containing extracellular vesicles [82]. In turn, educated mesenchymal stem cells release IL-6 associated with STAT3 activation and tumor progression with the formation of lung metastatic foci [83]. TGF-β exhibits higher expression in OS patients [84]. Based on these observations, combining anti-TGF-β antibodies and dendritic cells can enhance the systemic immune response and produce anti-tumor effects in OS. VEGF regulates the progression of tumors. Sunitinib, a VEGF receptor inhibitor, blocks VEGF signaling and reduces the accumulation of myeloid-derived suppressor cells that maintain the tumor microenvironment to evade immune responses [84]. Single-cell RNA sequencing revealed the high diversity of OS-associated mesenchymal stem cells and identified three main cellular clusters [17••] Even if the functional role of these cell subsets in the pathogenesis remains unclear, their gene expression feature already foreshadows their potential involvement in controlling cell differentiation, tumor angiogenesis, and metastatic processes. Such high heterogeneity is also applicable to tumor-associated osteoclasts and macrophages [17••]. By analogy with macrophage subsets, osteoclasts display phenotypic and functional plasticity with anti- or pro-inflammatory activity depending on their origin and the pathophysiological context [85]. Single-cell analysis confirmed the phenotypic heterogeneity in OS as well as in the primary tumor and in lung metastases [17••]. Tumor-associated macrophages recruited by OS cells are regulated by the local microenvironment, which is crucial for tumor development and metastasis initiation [73]. M2-polarized tumor-associated macrophages are associated with invasion and metastases in OS patients. The active derivation of vitamin A, all-trans retinoid acid (ATRA), induces cellular differentiation and arrests the proliferation of tumor cells through activation of transcription factors that regulate TAM polarization [86]. Inhibition of the M2 polarization of tumor-associated macrophages may restrain lung metastases in OS cells [86]. An inhibitory immune microenvironment that immunosuppressed regulatory T cells, myeloid-derived suppressor cells, and tumor-associated macrophages, is associated with the over-activation of pathways, including PD-1, IDO, TGF-β, STAT3, VEGF, and IL-10 [84]. In this context better characterization of the local tumor microenvironment of OS is mandatory for developing new targeted immunotherapies.

As previously mentioned, the pulmonary parenchyma is the main metastatic site of osteosarcoma cells. OS cells manipulate their local microenvironment but also prepare their distant pre-metastatic niche for hosting migrating cells through the release of extracellular vesicles [87]. IL-6 and CXCL-8 have been identified as key primary mediators of osteosarcoma tropism and their blockade prevents the formation of lung metastatic foci in preclinical models [88]. Similarly, ANGPTL2 [89] has been shown to be a key contributor in lung pre-metastatic niche formation. OS-derived extracellular vesicles that mediate the dialog between cancer cells and lung fibroblasts are responsible for fibroblast reprogramming and support metastatic progression [90]. Interestingly, myofibroblast reprogramming of osteosarcoma stem cells supports the establishment of lung macro-metastases [91]. In this context, anti-fibrotic agents represent new therapeutic options in osteosarcoma [92].

## New Strategies for Drug Delivery in OS

Reducing the toxicity and side effects of therapies and improving cancer cell targeting drug delivery systems have gained interest in OS in recent years.

### Nanoparticles (NPs)

The in vivo penetration and biocompatibility capacities of nanoparticles (NPs) have stimulated the interest of the scientific community regarding their use as drug delivery systems in oncology [93]. With the complexity of OS, radio-resistance and developing chemoresistance, NP drug-loading could result in innovative changes in OS [93]. Zinc oxide nanoparticles (ZnO NPs) are widely used in the biomedical field, such as antibacterials and sunscreen, thanks to their safe excipients [94]. Recently, it has been demonstrated that ZnO NPs inhibit the growth of several tumors, including OS [95, 96]. The growth inhibition of cell tumors mediated by ZnO NPs was mainly due to activation of HIF-1α/BNIP3/LC3B-mediated mitophagy in OS cells, inducing degradation of β-catenin, resulting in OS metastasis inhibition [94].

A third-generation bisphosphonate, zoledronic acid, induces tumor cell apoptosis, angiogenesis, and metastasis inhibition in vivo by arresting the S phase and through the DNA damage pathway [97]. Unfortunately, in contrast to preclinical models [98], clinical trial (ClinicalTrials.gov Identifier: [39]) did not demonstrate any beneficial effect of zoledronic acid combined with chemotherapy in OS patients [99]. The lack of therapeutic benefit in OS patients treated with zoledronic acid may be explained by the impact on macrophage polarization. The deregulation between M1/M2 populations may alter CD8^+^ T lymphocyte tumor infiltration [10, 100]. However, using bisphosphonates showed potential benefits as a drug delivery system to bone tumors with reduced toxicity. By binding to tumor-specific biomarker ligands, NPs can be used to deliver drugs to target tumors and may reduce the risk of adverse effects [20]. Similarly, hydroxyphosphonate-linked doxorubicin promoted strong antitumor effects with lower toxicity in preclinical models of OS and showed more potent activity than the doxorubicin/zoledronic acid combination [101].

OS cells are characterized by the expression of CD44 which is activated by its binding with hyaluronic acid (HA) and then participates in tumor progression, drug resistance, and metastasis [20]. Recent evidence shows that 100-nm inorganic NPs modified with HA and polyethylene glycol (PEG) on nano-hydroxyapatite (nHA) particles loaded with zoledronic acid can be used to target OS cells. HA-PEG-nHA-zoledronic acid inhibited OS proliferation in vivo by inducing a local inflammation which may lead to tumor necrosis without detectable systemic adverse effects [20].

### Liposomes

Liposome NPs were the first nano drug delivery systems used to protect in vivo the loaded drug from biodegradation [93]. In a phase I clinical trial (Trial registration number: ChiCTR1900021550), liposome-encapsulated doxorubicin appeared well tolerated with an acceptable safety profile in advanced OS [102]. A sialomycin-entrapped lipid-polymer with CD133 and EGFR aptamers was created to target CD133^+^ OS stem cells [103]. In this study, the authors showed that the polymer inhibited OS cell activity and in vivo tumor growth in OS-bearing mice. However, despite this anti-tumor activity, liposomes have low stability in serum in vivo, which can affect the immune response by macrophage phagocytosis and activate the complement pathway. This may limit their clinical application in OS [93].

### Hydrogels

The development of biomaterials and, specifically, bone scaffolds has become an excellent support for bone repair and drug delivery systems. Among the bone scaffolds, three-dimensional porous mesh gels with water absorbance, known as hydrogels, seem important [18, 93]. Hydrogels can be used as vehicles for therapeutic drugs. They can imitate the extracellular matrix, providing pharmacological treatment, growth and differentiation of mesenchymal stem cells, and improving bone regeneration [104]. Peng et al. showed that drugs encapsulated in hydrogels could significantly decrease OS volume in vivo in an Mg63-bearing mouse model. This result suggests that hydrogels can be injected locally at the tumor site with minimal invasiveness, good biosafety, and low systemic effects, and they can be used as drug delivery systems as single agents or in combined therapy [8]. Combining alendronate with oxaliplatin in a mPEG45-PLV19 thermosensitive hydrogel resulted in inhibition of the progression of OS and lung metastases in an in vivo mouse model [40].

Nano-hydroxyapatite (nHA) can also be used to inhibit tumor development [18]. nHA was embedded in a hybrid hydrogel light-induced photopolymerization, to mimic the extracellular matrix post-OS eradication and stimulate bone defect restoration in a murine OS model [105].

Polyvinylpyrrolidone iodine (PVP-1), an antibacterial agent, has become popular in anti-tumor treatment and is used as an irrigation fluid in cancer surgery to eliminate residual cancer cells and prevent local recurrent tumor growth [8]. A hydrogel of silk fibroin solution mixed with PVP-1 and meglumine diatrizoate (MD) was used in human OS cells in in vitro and in vivo experiments and induced the suppression of OS growth, leading to less systemic damage compared to chemotherapy [8].

While the physicochemical properties of hydrogels facilitate the control of drug delivery (e.g., reticulation), they do not show any mechanical resistance properties and may be associated with an increased risk of fractures and their inorganic nature with metal ions may also be associated with cytotoxicity [18]. Nevertheless, hydrogels are interesting drug delivery systems with real potential in the treatment of OS.

## Conclusion

In the absence of a significant increase in overall survival in OS in the last 4 decades, numerous new approaches are emerging. However, they will all require better characterization of the tumor microenvironment in spatial and temporal dimensions (e.g., the role of mesenchymal stem cell subsets in tumor progression that will require new technologies (e.g., digital spatial profiling, single cell RNA sequencing)). To decipher the key functions of each tumor microenvironment, compartments will make it possible to understand the local immune tolerance observed in OS although immune infiltrates are described in tumor tissues. OS is a heterogeneous tumor regulated by its microenvironment and counterproductively. The research in this field is carried out on 2D surfaces. Mimicking the 3D form of OS could lead to reproducible and applicable results [106, 107].

As bone and lung metastases are critical progression parameters, there is a clinical need to develop new tools for detecting residual and recurrent disease. Liquid biopsies with the detection of both circulating tumor cells and cell-free circulating tumor DNA (cfDNA) are two promising technical approaches and have the main advantage of not being very invasive, and thus more readily accessible for children. Circulating tumor cells detectable in the bloodstream can be released from the primary tumor site and metastatic nodules. They can be considered a snapshot of tumor heterogeneity at a given time and may have a strong value in longitudinal patient monitoring [108, 109]. Even if the lack of specific biomarkers expressed at the circulating tumor cell surface slows down their use in the clinical follow up of osteosarcoma, several recent works described the detection of circulating tumor cells in osteosarcoma [110–113]. Cell-surface vimentin [114] combined or not with GD2/3 detection [115] is a promising biomarker that may be used for isolating circulating tumor cells. cfDNA corresponds to DNA fragments released into the bloodstream by tumor tissues, modifying the associated tumor microenvironment [116]. Like circulating tumor cells, cfDNA reflects the genetic aberrations of osteosarcoma cells at a given time [117]. Interestingly, cfDNA detection was associated with inferior outcomes in osteosarcoma and thus appears to be a promising tool for disease surveillance in bone sarcoma [118, 119••, 120]. The recent literature showed the clinical potential of liquid biopsies, and large longitudinal clinical trials are mandatory for validating their use for prognostic or predictive biomarkers.

In light of the considerable heterogeneity of OS (e.g., high genetic mutation tumor burden, histological subtypes), individual tumor characterization will lead to the development of personalized therapies. OS has definitively entered the era of personalized medicine.
